# A Second Tubulin Binding Site on the Kinesin-13 Motor Head Domain Is Important during Mitosis

**DOI:** 10.1371/journal.pone.0073075

**Published:** 2013-08-28

**Authors:** Dong Zhang, Ana B. Asenjo, Michaela Greenbaum, Luping Xie, David J. Sharp, Hernando Sosa

**Affiliations:** 1 Department of Physiology and Biophysics, Albert Einstein College of Medicine, Bronx, New York, United States of America; 2 State Key Lab of Reproductive Medicine, College of Basic Medicine, Nanjing Medical University, Nanjing, Jiangsu, China; Consejo Superior de Investigaciones Cientificas, Spain

## Abstract

Kinesin-13s are microtubule (MT) depolymerases different from most other kinesins that move along MTs. Like other kinesins, they have a motor or head domain (HD) containing a tubulin and an ATP binding site. Interestingly, kinesin-13s have an additional binding site (Kin-Tub-2) on the opposite side of the HD that contains several family conserved positively charged residues. The role of this site in kinesin-13 function is not clear. To address this issue, we investigated the *in-vitro* and *in-vivo* effects of mutating Kin-Tub-2 family conserved residues on the *Drosophila melanogaster* kinesin-13, KLP10A. We show that the Kin-Tub-2 site enhances tubulin cross-linking and MT bundling properties of KLP10A *in-vitro*. Disruption of the Kin-Tub-2 site, despite not having a deleterious effect on MT depolymerization, results in abnormal mitotic spindles and lagging chromosomes during mitosis in *Drosophila* S2 cells. The results suggest that the additional Kin-Tub-2 tubulin biding site plays a direct MT attachment role *in-vivo*.

## Introduction

Kinesin-13s are MT depolymerases [1] that modulate MT dynamics during important cellular processes such as mitosis [2], cytokinesis [3], axonal branching [4] and ciliogenesis [5]. During mitosis, kinesins-13s locate at both ends of spindle MTs to regulate the size of the spindle and the movement of chromosomes toward the spindle poles during anaphase [6,7]. They are also involved in fixing incorrect MT-kinetochore attachments [8].

Like all kinesins, Kinesin-13s have a globular HD containing a microtubule and an ATP binding site. However, rather than binding to the MT lattice and producing a conformational change leading to unidirectional movement, the kinesin-13 HD preferentially binds to the MT ends where it induces the formation of curved tubulin protofilaments (PFs) [1,9]. This process involves binding to tubulin using the kinesin-13 family-specific elongated loop-2 as well as areas common to all kinesins [10]. We refer to this interface site as the putative kinesin-tubulin binding site or Kin-Tub-1. Interestingly, kinesin-13s have another tubulin binding site (Kin-Tub-2) on the other side of the HD that promotes the formation of kinesin-13-tubulin spirals and ring complexes around MTs *in-vitro* [11–13]. The presence of several kinesin-13 class-conserved residues in this area suggests that this binding site may also have an important physiological role *in-vivo*. To address this possibility, we investigated *in-vitro* and *in-vivo* the consequences of replacing key residues of the Kin-Tub-2 site on the *D. melanogaster* kinesin-13 KLP10A. Our results indicate that the unique Kin-Tub-2 site provides a MT attachment role independent of kinesin-13 MT depolymerization activity and that this binding site is important for proper spindle morphogenesis and poleward chromosome movement during mitosis.

## Results and Discussion

### Twin tubulin binding sites on the kinesin-13 HD bundle MTs and protofilaments

**Figure 1:**
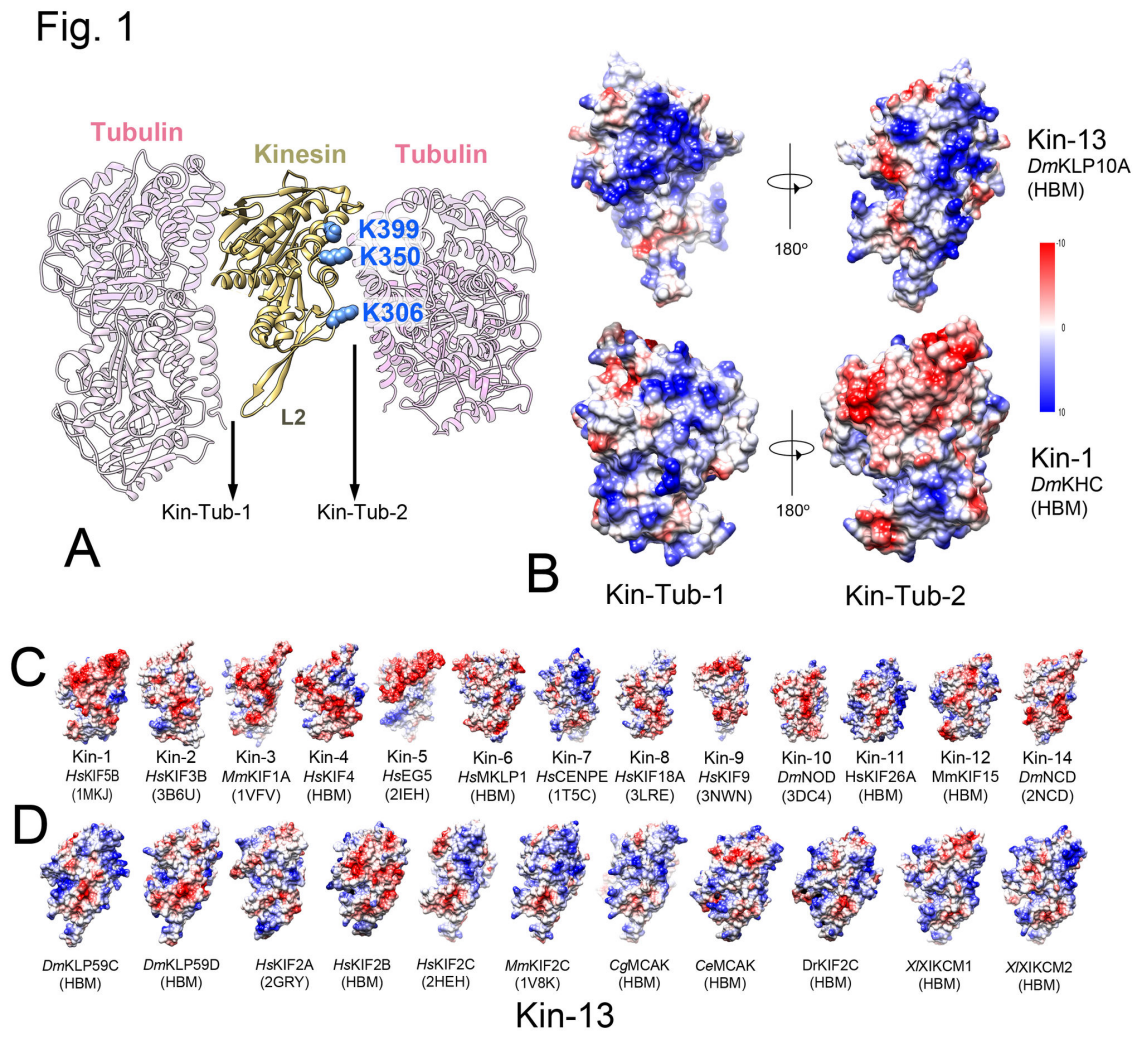
Kinesin HD electrostatic surface potentials in tubulin binding areas. (A) Ribbon representation of the KLP10AHD-tubulin-MT spiral complex model (PDB IC: 3J2U [10]) showing the two tubulin binding sites at opposite sides of the HD. The Kin-Tub-1 area corresponds to the putative MT binding site, common to all kinesins, but in this case it mediates binding to a curved tubulin protofilament. The Kin-Tub-2 area mediates binding of the kinesin-13-HD-curved-tubulin complex to the MT and the formation of spirals wrapping the MT. Mutating kinesin-13 class conserved positively charged residues in the Kin-Tub-2 area (KLP10A residues K306, K350, and K399 highlighted as blue atom spheres) disrupt these interactions and prevents the spirals from wrapping around MTs [13]. The location of the kinesin-13 Loop-2 (L2) is indicated. (B) Electrostatic surface potential comparison of Kin-Tub-1 and Kin-Tub-2 areas of a kinesin-1 (*Hs*KIF5B) and a kinesin-13 (*Dm*KLP10A). Color scale inset: -10 (red) to +10 (blue) kcal/mol·e. (C) Kin-Tub-2 area of several kinesin families. The corresponding kinesin family is indicated below each HD structure. (D) Kin-Tub-2 area of several kinesin-13s (all within the Kinesin-13B/MCAK subfamily [14]). In B-D the name corresponding to each kinesin sequence are indicated with an italics two letter prefix corresponding to the organism origin (*Ce: Caenorhabditis elegans; Cg: Cricetulus griseus; Dr: Danio rerio; Ds: Drosophila melanogaster; Hs: Homo Sapiens; Mm: Mus musculus; Xl: Xenopus laevis*) and the PDB IC below (references [46–52]. When no atomic structures were available, a homology based model (HBM) was calculated using the program Modeller [53].

In previous work we found that kinesin-13s can form oligomeric rings and spirals around MTs, mediated by an additional tubulin binding site on the HD that we called Kin-Tub-2 [13] (Figure 1A). A comparison of the electrostatic surface potential of the HD of several kinesins (Figure 1B–D) reveals that Kin-Tub-2 area of kinesin-13s is different from other kinesins. In most kinesins, this area is negatively charged, but in kinesins-13s, particularly in the 13B.MCAK/KIF2 subfamily [14], it tends to be positively charged. Some noted exceptions to this generalization are human KIF2B and *D. melanogaster* KLP59D, which are more electronegative in the Kin-Tub-2 site. It would be of interest to investigate whether this distinction is related to the common mitotic roles that these two kinesins play in human [6] and *D. melanogaster* cells [15].

Electrostatic interactions are important for kinesin and many other proteins that bind to MTs [16–19]. The MT surface has an overall negative electrostatic potential [20] and the binding sites of many MT associated proteins are positively charged (e.g. see kinesin Kin-Tub-1 site, Figure 1B). The fact that many kinesin-13s are distinctly electropositive on both sides of the HD suggests that they are adapted to interact with tubulins at either side of the HD. Such adaptation may contribute to MT attachment and bundling at cellular locations where kinesin-13s are concentrated, such as mitotic spindle poles or kinetochores [7,21–24]. It is interesting to note that another case of a positively charged Kin-Tub-2 site is found on the Kinesin-7, CENPE (Figure 1C), which is thought to have a MT attachment role and link chromosomes to spindle MTs [25–27].

**Figure 2:**
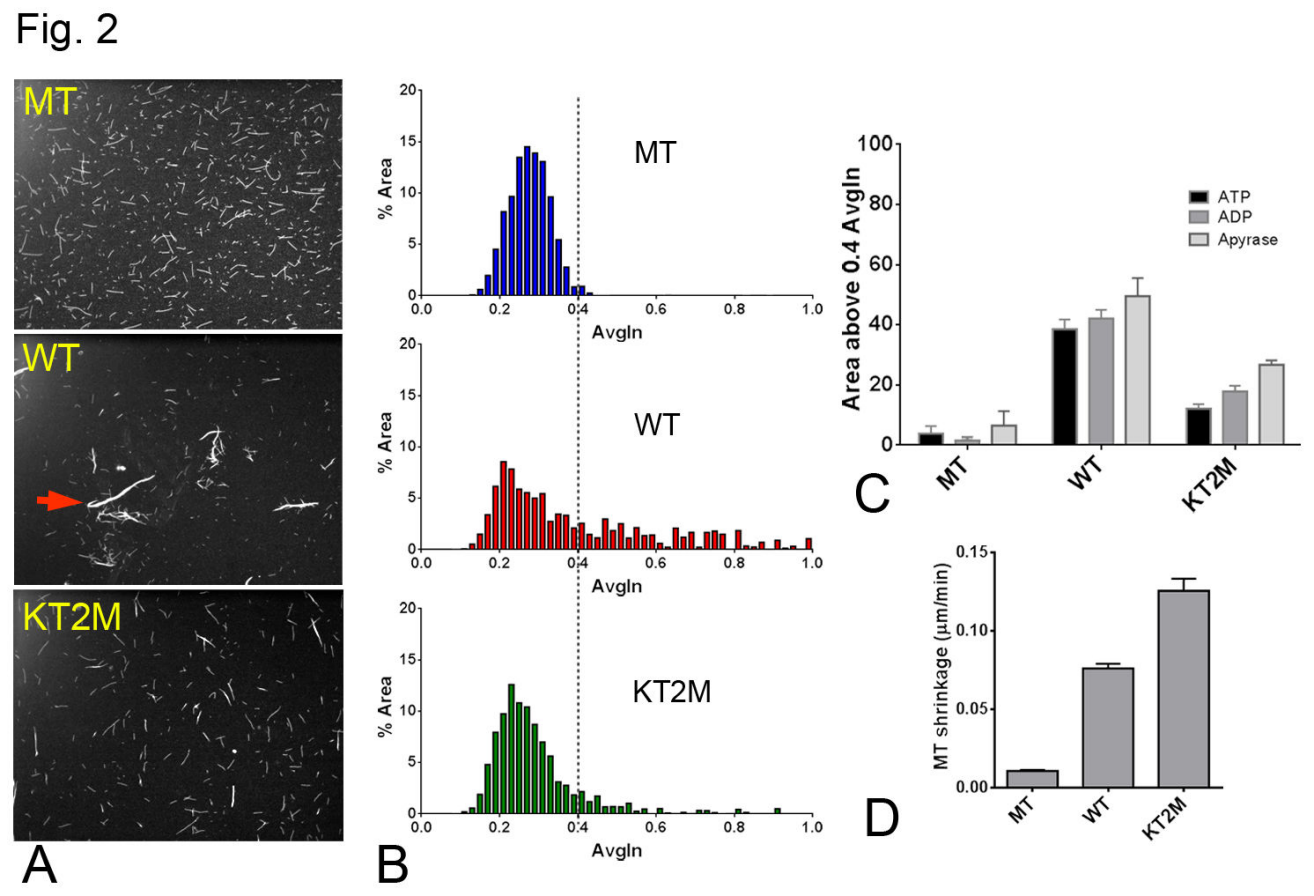
In-vitro bundling and depolymerization assays. (A) Fluorescent MTs images. Top: MTs without KLP10A; Middle: MTs incubated with KLP10AHD. Red arrow points to a bundle example; Bottom: MTs incubated with mutant KT2M- KLP10AHD. (B) % Area vs. Average fluorescence histogram, (C) MT bundling activity bar plot. The total number of slide images analyzed (N) is 9 for each MT-only condition and 24 for each WT and KT2M conditions. (D) MT shrinkage rate. The total number of microtubules analyzed (N) to estimate the MT shrinkage rate in D is 104,179 and 176 respectively for MT, WT and KT2M.

To investigate whether the kinesin-13 HD and its Kin-Tub-2 site promotes attachment and interaction between MTs, we devised an *in-vitro* fluorescence MT bundling microscopy assay. In this assay, bundles containing more or fewer MTs were distinguished by their size (area in the micrograph field) and relative fluorescence intensity. We comp**a**red the MT bundling activity of KLP10AHD constructs with or without mutations in the Kin-Tub-2 site. Figure 2A shows representative fields of: 1) MTs in the absence of KLP10A; 2) MTs incubated with wild type (WT) KLP10AHD; and 3) MTs incubated with a KLP10AHD construct in which three lysines on the Kin-Tub-2 site were replaced by alanines (Kin-Tub-2 mutant or KT2M). The residue replacements in (3) prevent the formation of KLP10A-tubulin oligomeric spirals around MTs *in-vitro* [13] (Figure 1A). In the absence of KLP10A, most filaments appear shorter and with less fluorescence intensity (Figure 2A, top) than when incubated with KLP10AHD where the presence of many large bundles was clear (Figure 2A, middle panel). An increase in bundling activity was also observed with full-length KLP10A constructs (not shown). Bundling activity was quantified by selecting all the separate filament regions (region of interest) in the images, calculating the area of each region (number of pixels) and their average fluorescence intensity. We then plotted the fraction of the area corresponding to each average intensity value (Figure 2B). Images with more bundles would have more values with higher average intensities (toward the right of the frequency distributions). In the absence of KLP10A, MTs produce a Gaussian distribution with few values over 0.4 relative intensity units. The presence of KLP10A results in more areas with higher average intensity values indicating increased bundling (Figure 2B). Figure 2C shows the fraction of areas with intensity values over a 0.4 intensity threshold for each experimental condition. We used this criterion to quantify and compare bundling activities. The presence of KLP10AHD produces a very significant increase in MT bundling activity (P << 0.01) with small differences (P>0.05) related to the particular adenine nucleotide present in the experiment. We also observed a very significant (P << 0.01) decrease in MT bundling activity for the KLP10AHD KT2M construct relative to wild type, indicating that the Kin-Tub-2 site specifically enhances the ability of KLP10A to bundle MTs. As in a previous study [13], we found that Kin-Tub-2 mutations did not impair the ability of KLP10A to depolymerize MTs (Figure 2D, Movie S1). The relatively higher depolymerization rate observed for the mutant KT2M construct may result because the formation of MT rings and bundles inhibits depolymerization.

**Figure 3:**
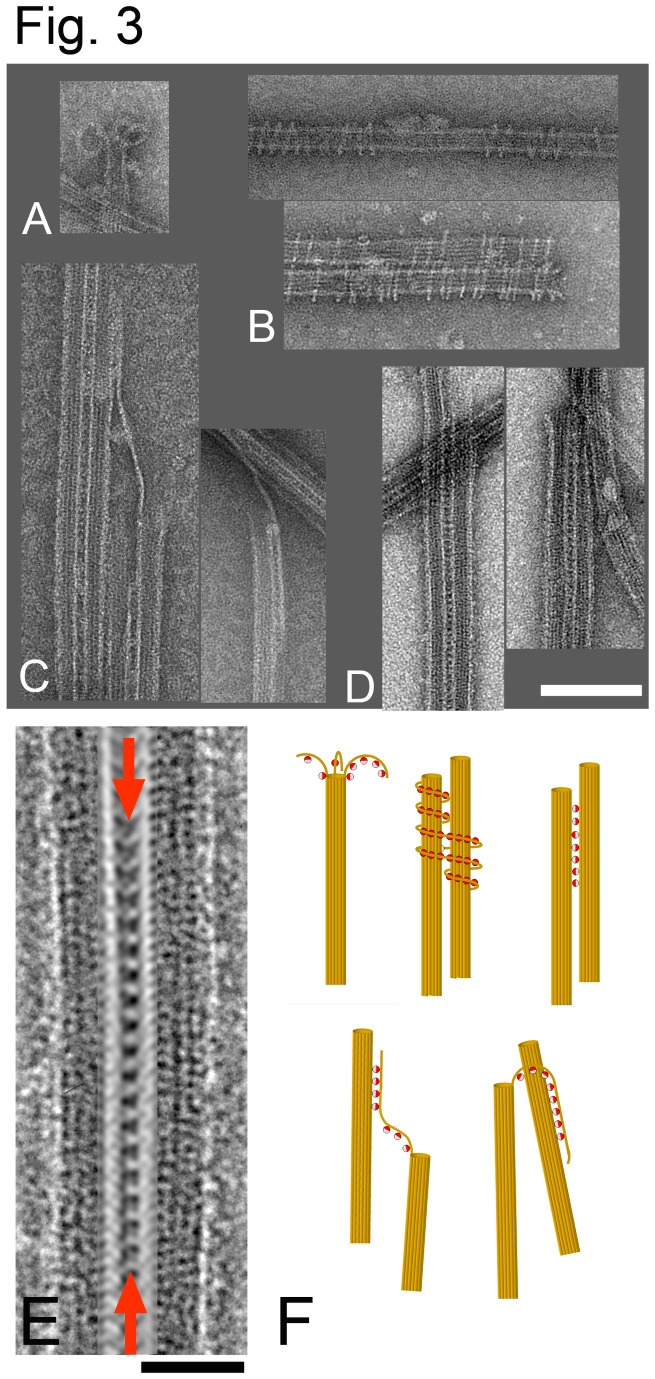
EM of KLP10AHD induced bundles. (A–D) Gallery of representative EM structures observed when incubating KLP10AHD and MTs. (E) Image of two MTs cross-linked by KLP10AHD molecules (indicated by the red arrows). The area connecting the two microtubules was enhanced by applying a 1/8 nm^-1^ layer line filter in Fourier space. (F) Interpretation of the observed EM structures. MTs and PFs in ochre; KLP10AHD represented as two color spheres, half red (Kin-Tub-1 side) and half pink (Kin-Tub-2 side).

To further understand the bundling mechanism, we examined KLP10AHD-MT bundles by electron microscopy (EM). We concentrated our analysis on bundles with few MTs where the connections between MTs could be most easily discerned. However, numerous bundles with many MTs superimposed (not shown) were also observed. A gallery of representative micrographs is shown in Figure 3A–D. All images correspond to MTs incubated with the KLP10AHD WT construct in the presence of ATP, except the one in the bottom of panel 3B that was obtained in the presence of the non-hydrolysable ATP analogue AMPPNP. Panel A shows an example of the typical depolymerization rings observed at MTs ends when MTs are incubated with KLP10A in the presence of ATP [10]. Panel 3B shows examples of the spirals and rings that kinesin-13 constructs form around MTs. Interestingly, we observed the formation of these spirals in the presence of ATP (Figure 3B top), although with less frequency than in the presence of AMPPNP. The fact that these oligomeric rings can also form in the presence of ATP, the natural kinesin substrate, suggests that similar rings could be formed in physiological conditions during MT depolymerization. Note also that the long single PFs observed are not an artifact due to the presence of Taxol or other MT stabilizing agents as they have also been observed in the absence of any tubulin drug [10]. Panel 3C show examples of MTs linked to other MTs through a PF and panels 3C and 3D MTs cross-linked with what appears to be KLP10AHD molecules regularly arranged between MTs. The molecules bridging MTs are regularly spaced every ~8 nm as is expected from kinesin decorated MTs. However, different from other kinesins, KLP10AHD decoration is concentrated to the area between MTs instead of decorating the whole tubulin lattice of each MT. This decoration pattern suggests an increased KLP10A affinity for MTs when both tubulin binding sites can be occupied. Figure 3E shows the area between MTs after applying a filter to reduce noise. The filtered image shows what appears to be a row of KLP10AHD molecules bridging the two MTs, consistent with a model in which a KLP10AHD molecule cross-link two adjacent MTs through Kin-Tub-1 interactions to one MT and Kin-Tub-2 interactions to the other. Figure 3F shows a summary of possible KLP10AHD interactions leading to MT bundling, based on the observed EM structures.

### Disruption of the Kin-Tub-2 interface of KLP10A causes mitotic defects in D. melanogaster S2 cells

**Figure 4:**
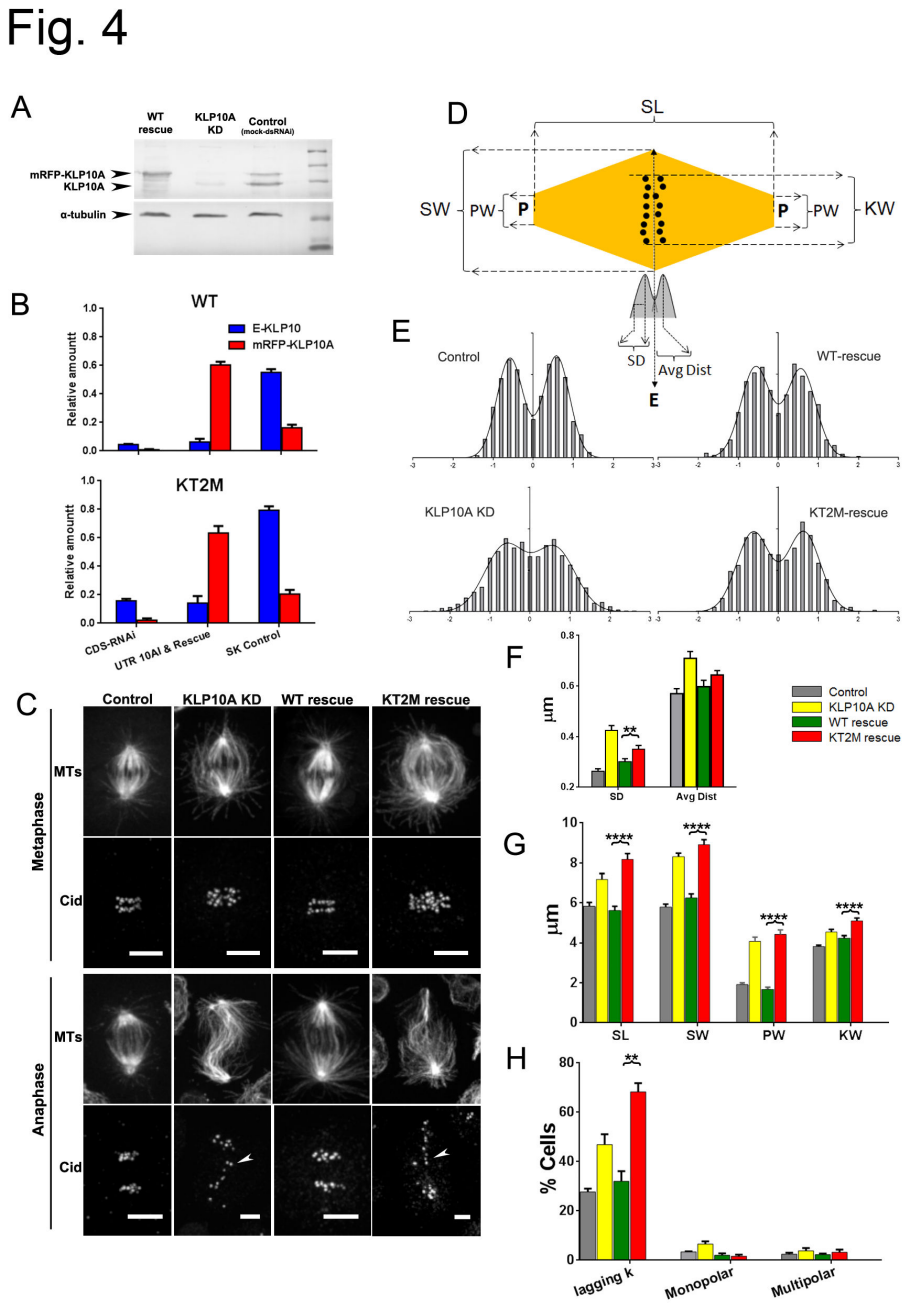
Effect of KLP10A Kin-Tub-2 mutations on S2 cells during mitosis. (A) Western blot of cell line transfected with mRFP-WT-KLP10A (WT cell line). Lanes: WT-rescue: Cells after dsRNAi treatment against endogenous KLP10A untranslated RNA region and induction of exogenous mRFP-WT-KLP10A expression; KLP10A KD: dsRNAi treatment against KLP10A coding sequence; Control: mock dsRNAi treatment; No-label: molecular weight markers ladder. (B) Relative amount of KLP10A proteins in cell lines containing plasmids for mRFP-WT-KLP10A (top) and mRFP-KT2M-KLP10A (bottom) (N=3). (C) Fluorescent images (GFP-α-tubulin) of representative mitotic spindles. (D) Explanation of evaluated spindle metrics. P: pole, E: equatorial plane. SL: spindle length; SW: spindle width; KW: kinetochore area width; SD: standard deviation of the distribution of kinetochore distances to the equatorial plane. AvgDist: mean distance of kinetochores to the equatorial plane. (E) Distribution of metaphase kinetochore positions for all the cells analyzed (31 in each case). (F) Kinetochore distributions mean SD and AvgDist (N=31). (G) Mean SW, KW and SL (N = 39, 42, 39, 41 respectively for Control, KLP10A-KD, WT-rescue, KT2M-rescue). (H) Mean frequency (%) of cells with lagging kinetochores during anaphase-A, monopolar and multipolar spindles (Number of repeat experiments, N=3 for each case).

The results of the previous section indicate that the unique Kin-Tub-2 site of kinesin-13 has a significant influence on the way that kinesin-13 interact with MTs *in-vitro*. However, whether these interactions are important for kinesin-13 function *in-vivo* is not clear. To investigate whether the positively charged kinesin-13 Kin-Tub-2 site has functional importance *in-vivo*, we used a knock-down and rescue approach to compare cells during mitosis with or without mutations in the Kin-Tub-2 site of KLP10A. We generated *D. melanogaster* S2 cells lines transfected with plasmids coding for KLP10A labeled with monomeric red fluorescent protein (mRFP) under the control of a copper-inducible promoter (methods). Two cell lines were made containing plasmids with either the KLP10A WT sequence (WT cell line) or KLP10A with the same Kin-Tub-2 residue changes as the KT2M mutant described in the previous section (KT2M cell line). The relative amounts of endogenous and exogenous KLP10A in the cell lines after distinct dsRNAi treatments and protein expression induction regimens were estimated by western blot analysis (Figure 4A, B). Figure 4C shows representative mitotic spindles images of fixed cells during metaphase and anaphase. Control corresponds to cells treated with dsRNA against no *D. melanogaster* protein (mock dsRNAi treatment); KLP10A-KD to cells treated with dsRNA against the KLP10A coding sequence (i.e. targeting endogenous and exogenous KLP10A expression). The phenotype of cells after mock dsRNA treatment was similar for WT and the KT2M cell lines so data from these two cell lines were pooled together under a single control category. For the same reason, data from the two cell lines dsRNA treated against the coding sequence of KLP10A were pooled together under a single KLP10A-KD category. WT-rescue and KT2M-rescue correspond to cells dsRNA treated against KLP10A mRNA untranslated regions (UTR, i.e. targeting only endogenous KLP10A expression) followed by induction of exogenous KLP10A expression. In the WT-rescue and KT2M-rescue cells, exogenous KLP10A (WT or KT2M) represents more than 83% of the total KLP10A expressed (Figure 4B). Therefore, differences between these two cell groups can reveal cellular processes where the positively charged Kin-Tub-2 site plays a role.

Relative to controls, the mitotic spindles of KLP10A-KD cells were longer and wider with many MTs reaching regions outside the area where the kinetochores are located (Cid protein labeled). The kinetochores also appeared more misaligned relative to the spindle equator. During anaphase the spindle shape became more distorted and there was an increase in the number of lagging chromosomes as indicated by the very misaligned kinetochores (Figure 4C). These results are consistent with previously described defects in Drosophila cells after KLP10A inhibition [7] or knockdown [28,29]. To quantify these observations, we took several spindle metrics from many mitotic cells (Figure 4D–G) and estimated the relative amount of cells with lagging chromosomes, and monopolar or multipolar spindles (Figure 4H). We found several statistically significant differences between the WT-rescue and the KT2M-rescue cells. The distribution of kinetochore positions in the direction perpendicular to the spindle equator is wider for the KT2M-rescue relative to the WT-rescue cells. The spindle length (SL), width (SW) and pole width (PW) are larger for the KT2M-rescue cells than for the WT-rescue and even slightly larger than in the KLP10A-KD cells (Figure 4G). The kinetochores of the KT2M-rescue cells also have a small but significant increase in the width of the kinetochore distribution in the direction parallel to the spindle equator (KW in Figure 4 G) relative to the other three experimental conditions. The number of cells in anaphase with lagging chromosomes is also significatively higher for the KT2M-rescue cells than for the cells in the other three experimental conditions (Figure 4H). We did not detect significant differences in the number of cells with multipolar spindles (Figure 4H). Knockdown of KLP10A (KLP10A-KD) produces an increase in the relative number of cells with monopolar spindles relative to controls but the WT-rescue, as well as the KT2M-rescue, were not significatively different from controls.

In summary, expression of exogenous WT KLP10A (WT-rescue) rescues the KLP10A knockdown (KLP10A-KD) phenotype but the KT2M mutant (KT2M-rescue) failed to produce a full rescue and exacerbated some of the knockdown defects. These results indicate that the Kin-Tub-2 site of KLP10A has an important role for the normal progression of mitosis in *D. melanogaster* S2 cells.

### Disruption of the Kin-Tub-2 interface of KLP10A does not affect poleward flux

**Figure 5:**
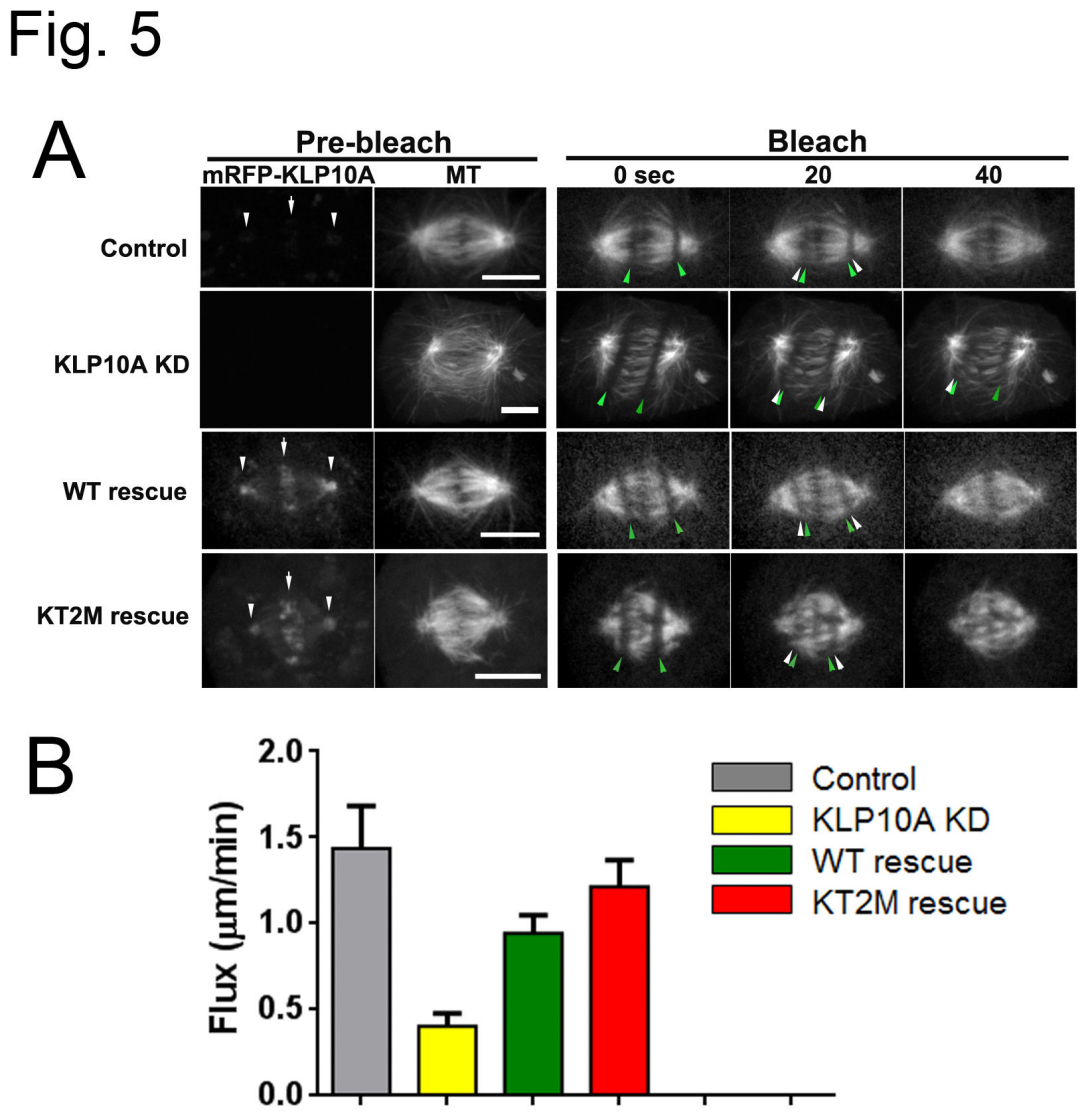
Poleward flux rate and KLP10A-MT pole attachment models. (A) Fluorescent live cell images before and after a bleaching beam line was flashed to each side of the mitotic spindle. Poleward flux causes the bleach mark to move towards the spindle poles. The original and current positions of the bleach mark front are indicated by the green and white arrows respectively. Side by side pre-bleach images of the GFP-α-tubulin and mRFP channels are shown. Only the GFP-α-tubulin channel was recorded in the time series after the bleaching flash. (B) Mean poleward flux rates (N=60, 72, 52, 40 respectively for Control, KLP10A-KD, WT-rescue, KT2M-rescue).

The rate of MT depolymerization at the spindle poles, which drives tubulin poleward flux, is modulated by kinesin-13s and, depending on the cell type, can be a major contributor to anaphase-A chromatid to pole movement [30]. In *Drosophila* cells, KLP10A inhibition results in an almost complete suppression of poleward flux [31]. To assess whether Kin-Tub-2 mutations affect the ability of kinesin-13s to depolymerize MTs *in-vivo*, we measured the anaphase-A poleward flux rates of the cell groups described in the previous section using fluorescent live cell imaging (Figure 5, Movie S2). Figure 5A (left) shows side by side mRFP (KLP10A) and GFP (tubulin) channels of mitotic spindles before the onset of anaphase. After induction of mRFP-KLP10A expression either in WT or the KT2M mutant, the protein concentrates at spindle poles and kinetochores, as has been previously described for endogenous and GFP labeled KLP10A [7,21]. KLP10A reduction (KLP10A KD) produces significant decrease of poleward flux rate relative to control (Figure 5B) as previously reported [7,32]. Exogenous expression of mRFP-KLP10A rescues the poleward-flux rates but the values were not significatively different whether the exogenous protein expressed was wild type or the Kin-Tub-2 mutant (WT-rescue vs. KT2M-rescue). These results indicate that disruption of the Kin-Tub-2 site does not interfere with KLP10A localization to the mitotic spindle or with the poleward flux-rate. These are not unexpected results considering that KT2M mutations do not inhibit MT depolymerization activity *in-vitro* (Figure 2D) and that residues important for cellular localization of KLP10A and other kinesis-13s are found outside the HD [33–36]. On the other hand, the mitotic defects associated with the KT2M mutations (previous section) cannot be attributed to KLP10A mislocalization or impaired depolymerization activity. We instead propose that kinesin-13s have a MT binding role associated with their unique Kin-Tub-2 binding site and that this role is independent of MT depolymerization activity. Disruption of the Kin-Tub-2 site would impair MT binding and produce the phenotype observed in KT2M-rescue cells.

### Models for KLP10AHD mediated MT attachment to spindle poles

**Figure 6:**
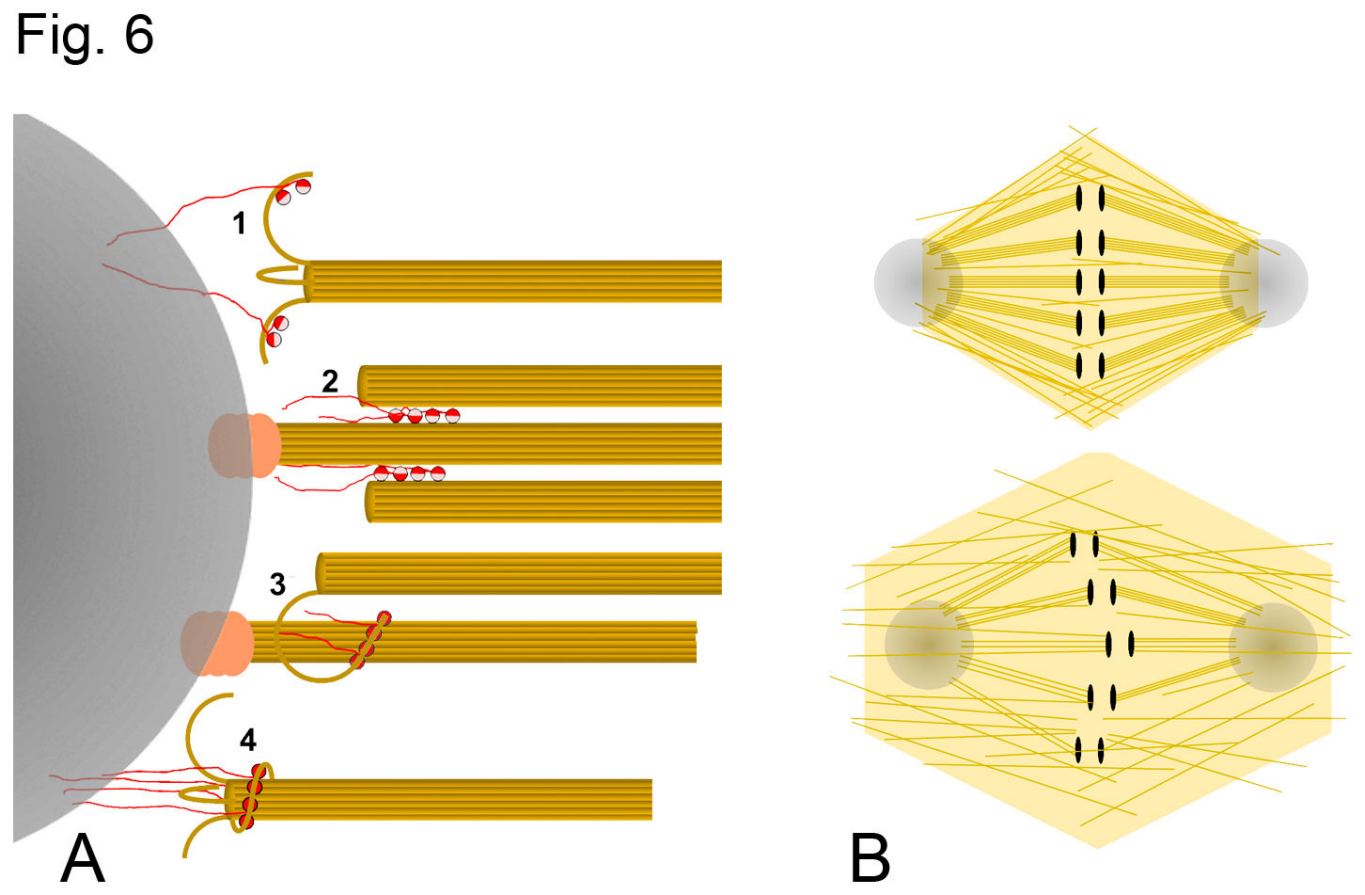
KLP10A mediated MTs pole attachment models. (A) Four possible KLP10A mediated MT pole attachment models based on the structures observed by EM (Figure 3). 1: Multiple pole-attached KLP10As depolymerize and keep the MT end attached to the pole. 2: Multiple KLP10As crosslink pole-unattached MTs to pole-attached ones. 3: Pole-unattached MTs interact with pole-attached ones through PFs with multiple KLP10As bound. 4: Ring of pole attached KLP10As and tubulin form a sleeve around a MT attaching it to the pole. All the MT-pole attachment models proposed could coexist together and analogous mechanisms could contribute to MT-kinetochore attachments. Structures resembling model 1 has been observed in the kinetochores of mammalian cells [54]. (B) Model to explain observed KLP10A KT2M rescue phenotype of S2 cells. Top: In control and WT-rescue rescue cells the MTs comprising each kinetochore fiber (K-fiber) are tightly bundled and attached to poles and kinetochores. Bottom: In KT2M-rescue cells the altered Kin-Tub-2 binding site of KLP10A results in the detachment and separation of MTs from the K-fiber bundles. Random uneven loss of MTs from sister K-fibers results in kinetochore misalignments. The MTs separated from poles and kinetochores lead to the appearance of a larger and less organized spindle. Red/pink spheres: KLP10AHD; Red strings: KLP10A N and C terminal tails; Ochre cylinders and lines: MTs, Orange blob: MT capping proteins; Gray circles: spindle pole matrix in gray; Black ellipses: kinetochores.

The observed phenotypes of the KT2M-rescue cells can be accounted by models in which multiple kinesin-13 tubulin binding sites keep MTs close together and attached to the spindle pole (Figure 6A). In these models pole-bound-Kinesin-13s interacting with MTs using either of their two HD binding sites would keep MTs attached to the poles, even while they are adding or loosing tubulin subunits from their ends. The models are analogous to the type of dynamic attachments that some kinetochore proteins are thought to keep with the MT plus end. Many kinesin-13 HDs could mediate weak interactions with the MT that would keep a grip on the MT end as in a modified Hill type mechanism [37,38]. In addition, the fact that kinesin-13s can form oligomeric rings encircling MTs (Figure 3B) suggests that mechanisms analogous to the one proposed for the yeast Dam1 ring complex [39,40] could also be at play. A direct MT attachment role for Kinesin-13s is supported by i*n-vitro* assays indicating that kinesin-13s are able to hold the end of a depolymerizing MT under tension [41].

The proposed MT attachment models (Figure 6A) predict that disruption of the Kin-Tub-2 binding site would lead to the detachment of some MTs from the poles (Figure 6B). These detached MTs away from the poles could either become longer by polymerization or being pushed past the poles by kinesin-5 activity [42] resulting in longer, wider and less focused spindles as observed in the KLP10A-KT2M rescue cells (Figure 4G). Detachment of spindle microtubules form the poles would also result in an altered balance of poleward and anti-poleward forces leading to kinetochore misalignments during metaphase and lagging chromosomes during anaphase as observed (Figure 4E,F,H).

The observed KT2M phenotype could also be the result of a disrupted interaction between some unknown KLP10A cellular regulatory factor and the Kin-Tub-2 site. However, in the absence of a candidate regulator and with the available evidence for a specific tubulin interaction, we currently favor models involving only interactions with tubulin. Regardless the mechanism involved the KLP10A knockdown and rescue experiments clearly indicate that the Kin-Tub-2 site is important for kinesin-13 function *in-vivo*.

The fact that kinesin-13s form homodimers *in-vivo* [43] raises the question of what would be the added functionality of having the two tubulin binding site on a single HD instead of a single site on each of the two HDs. One possible explanation is that what is required is the ability to bind simultaneously to a curved PF through the Kin-Tub-1 site and to a straight tubulin through the Kin-Tub-2 site, as shown in models 3 and 4 of Figure 6A. There is also evidence that the two heads of a kinesin-13 dimer interact with two adjacent protofilaments in a microtubule [44]. This would render them unable to cross-linking two different MTs (or protofilaments from different MTs) without an additional tubulin binding site.

KLP10A is concentrated at the spindle poles, but it is also present at the kinetochores [7] (Figure 5A). Thus, the proposed MT attachment models may be also applicable at the kinetochores. These kinesin-13s mediated attachments would be in addition to other proteins complexes shown or thought to link MT to poles or kinetochores [45]. Besides adding redundancy, the additional MT binding site on the kinesin-13 HD would facilitate concurrent microtubule attachment and depolymerization activities.

## Materials and Methods

### Construction of KLP10A plasmids and mutagenesis

Drosophila melanogaster KLP10A head domain (residues I279 to I615) was bacterially expressed and purified as previously described (Tan et al., 2006; Tan et al., 2008). Mutations were introduced into the plasmid vector (pRSETB) using the QuikChange Lightning Multi Site-Directed Mutagenesis Kit (Agilent Technologies). The triple mutation KT2M consists of the following KLP10A residue substitutions: K306A, K350A, and K399A (Figure 1A). Full length KLP10A was cloned into pFastBacHTA (Invitrogen), recombined into bacmid, baculovirus expressed and purified as described [55]. For inducible cell expression of KLP10A, mRFP was fused to N-terminus of full-length KLP10A (mRFP-KLP10A) and cloned into pMT/V5-HisC (Invitrogen) and transfected into S2 cells as described [33], The expression of mRFP-KLP10A can be induced by the CuSO4 because the pMT/V5-HisC has metallothionein promoter (pMT) upstream of mRFP-KLP10A.

### Microtubule bundling assay

10 ul mixtures of rhodamine labeled microtubules (1.5 μM) and KLP10 HD (0.15 μM) were incubated for 10 minutes at room temperature in BRB80 buffer supplemented with 20µM Taxol, 1mM DTT, and either 2mM ATP, 2mM ADP or Apyrase (5 U ml-1). The incubation mixture was then diluted 22.5 fold, flowed into a 22mm x 22mm cover-slip glass flow chamber and let it sit for 4 min. The bottom cover-slide was DETA-treated to improve MT binding [56]. After brief wash with BRB80, supplemented with 20µM Taxol, the mixture was fixed by flowing a 1% glutaraldehyde solution in BRB80 and incubating for 3 min followed by cold methanol at -20^°^C. After a brief wash with BRB80, mounting medium (5% n-propyl gallate, 50% glycerol, 100 mM Tris-HCl, PH 7.5) was flowed in to prevent photobleaching. The fixed MTs in the chamber were imaged in a confocal microscope (Ultraview, PerkinElmer) using 561 wavelength laser excitation. Confocal images of areas (65 x 65 μm^2^ and 6 Z-section of 0.25 µm each) were collected and analyzed with ImageJ [57] and custom written Python scripts. Briefly: Images were segmented into separate regions of interest (ROIs) contain MTs using the "threshold" and "analyze particles" routines of imageJ. The number of pixels and average fluorescence intensity of each ROI was then recorded and used to build average fluorescence intensity distributions for each experimental condition (Figure 2B).

### In vitro microtubule depolymerization assay

Depolymerization assays were performed in 22mm x 22mm cover-slip glass flow chambers. The following solutions were sequentially flowed into the chamber; 1) Kinesin-1 rigor mutant (G234A, 0.15mg/ml, 4 min incubation) to promote MT attachment to the glass surface. 2) Blocking solution (7.5mg/ml BSA, 4 min incubation) 3) Rhodamine labeled microtubules in BRB80 solution (80 mM PIPES, 1 mM EGTA, 1 mM MgCl2, pH 6.9) supplemented with 20µM Taxol. 4) KLP10A (WT or K2TM to be tested) solution 30 nM KLP10A in 12 mM Pipes, 2 mM MgCl2, 1 mM EGTA, pH 6.8 7.5mg/ml BSA, 1mM DTT, 2mM of ATP, ADP or 5 U ml-1 apyrase, 220 μg ml−1 glucose oxidase, 22.5 mM glucose, 36 μg ml−1 catalase). In each case control experiments were performed by flowing the same solution without KLP10A. MTs in the flow chamber were imaged and movies recoded on an inverted Ultraview spinning-disc confocal system confocal microscope (PerkinElmer) using 546 nM laser excitation at 800 ms exposure, 10 sec interval and 0.25µm z-steps. Sample z thickness at each time point was always within 0.7µm. From the resulting movies MTs shrinkage rate was estimated by measuring the lengths of many MTs at 0 sec and 400 sec after flowing KLP10A or control solutions. All experiments were done at room temperature.

### Electron microscopy

Polymerized tubulin (3 μM) was incubated with WT KLP10 HD (3 uM) in the presence of 2 mM ATP or 2 mM AMPPNP (in BRB80, 2 mM MgCl2 solution) for 5-15 min at room temperature. 4 ul of the incubation mix were placed on freshly UV discharged 400 mesh carbon coated grids for 4 min and the grids were then negatively stained with 1% uranyl acetate. Electron micrographs were recorded at 120 kV on a FEI Tecnai-20 electron microscope equipped with a 2Kx2K TVIPS F224 HD digital camera at a nominal magnification of 50KX.

### Cell lines, double stranded RNA interference (dsRNAi) and exogenous KLP10A induction (Rescue)

Wild type (WT) S2 cells were obtained from ATCC (Manassas, VA). Transgenic S2 cell lines were made by transfecting WT S2 cells with plasmids containing GFP-α-tubulin and mRFP-KLP10A (wild type or KT2M triple mutant) with Effectene transfection reagent (Qiagen) and selected with 300 µg/ml hygromycin and 40 µg/ml blasticidine (InvivoGen). S2 cell culture and RNA interference treatment with double-stranded RNA (dsRNAi) was done as previously described [58]. DsRNA was synthesized and purified by T7 RiboMAX™ Express Large Scale RNA Production System (Promega), so all primers for dsRNA templates were composed of the T7 polymerase promoter sequence (5-TAATACGACTCACTATAGGG-3) appended to the 5′end of the specific KLP10A sequences. Primers for dsRNAi against a KLP10A coding sequence region are forward, 5′- CATGATTACGGTGGGGCAG -3′, and reverse, 5′- CAGCTCCCTGGGTTGTGG-3′. Primers for dsRNAi against KLP10 untranslated mRNA regions (UTR) were as in [21]. Primers for control dsRNAi were made against 900 bp of noncoding sequence from pBluescript SK [15].

For each dsRNAi and rescue experiment, 1 million double transgenic S2 cells (GFP-tubulin and mRFP-10A) were seeded into 6-well plate (35 mm each in diameter) and treated with 20 μg dsRNA on day 0 and day 2, 40μg dsRNA on day 4. On day 5, CuSO4 was added into the media to a final concentration of 0.25 µM for 4 hours to induce the expression of exogenous mRFP-KLP10A to a level close to endogenous KLP10A in control cells. Cells were then fixed right away for immunofluorescence. For live cell imaging, CuSO4 was diluted to 0.1µM after 4-hour induction with 0.25 µM CuSO4 to maintain a steady expression of exogenous KLP10A.

### Immunofluorescence microscopy

S2 cells were plated on concanavalin-A-coated coverslips for 4 h, fixed in 100% methanol at −20 °C for at least 20 min and blocked with 5% normal goat serum in PBS containing 0.1% Triton X-100. Primary antibodies used: rabbit anti Dm-KLP10A [7]; Chicken anti Cid, (gift from Dr. Gary Karpen lab); mouse anti α-tubulin (DM1A, NeoMarkers, Fremont, CA); Primary antibodies were applied at 1–20 μg ml−1 final concentration in blocking buffer. Fluorescent secondary antibodies (Jackson Immuno-Research Laboratories) were used at 7.5 μg ml−1. Imaging was carried out on an Ultraview spinning-disc confocal system (PerkinElmer) mounted on an inverted microscope (Eclipse TE300; Nikon) with a 100× (1.4NA) or 60× (1.4NA) objective and captured with an Orca ER digital camera (Hamamatsu).

### Mitotic spindle measurements

Immunofluorescence images Z-stacks of cells in mitosis in each experimental condition were selected to take several spindle dimension metrics (Figure 4D). The mitotic stage of the fixed cells was determined by the kinetochore positions. Cells in metaphase with a clear metaphase plate with aligned kinetochores forming two nearly parallel lines separated by less than ~2 μm (metaphase plate) were selected. The Z-slices containing a complete spindle were projected into a 2D image where length measurements were done manually with ImageJ measure tool. Kinetochore position distributions in metaphase were determined from the positions of the anti-Cid fluorescent peak maxima. A custom Python script was used to read the XY kinetochore positions of each cell and: 1) Determine the position and orientation of the spindle equator line by fitting a regression line to all the kinetochore positions. 2) Determine the perpendicular distance of each kinetochore to the equator line and the average & SD of the distribution of these distances. 3) Align the kinetochore distributions of several cells within a group by rotating and displacing each kinetochore distribution to a common centroid point and spindle equator line. Cells were scored as lagging chromosomes if at least one kinetochore was lagging behind at late anaphase (vs. all kinetochores being at the spindle poles).

### Live-cell imaging and tubulin poleward flux measurements

To measure microtubule flux in metaphase spindle, two thin rectangular regions of interest (ROI) to be photo-bleached were drawn in the middle of each half spindle and parallel to the spindle equator. Photobleaching was executed for 300ms for 4 times, then four-dimensional time-lapse movies of GFP–α-tubulin were acquired using the 488 laser of Ultraview spinning-disc confocal microscope at 300 ms exposure, 5 sec interval and 1µm z-step, sample z thickness at each time point was always within 3µm. The resulting movie files were opened in ImageJ to estimate tubulin-poleward flux rates. A thin line was drawn along the spindle long axis of each cell and a kymograph was generated with the ImageJ "reslice" function. Poleward flux rate was determined from the angle of the bleach line in the kymographs as done previously [59].

### Data analysis

Data plots and t-test between data sets were performed using Prism v. 6.08 (GraphPad Software Inc.). When indicated the statistical significance (P-value) between data sets pairs is noted according to: *, P ≤0.05; ** P≤ 0.01; ***, P ≤ 0.001; ****, p≤0.0001. Unless noted error bars always represent the standard error of the mean.

## Supporting Information

Movie S1 **Depolymerization of rhodamine-labeled, taxol-stabilized MTs in the presence of (from left to right): Control solution (without KLP10A); 30 nM WT-KLP10AHD, 30 nM KT2M-KLP10AHD.** Total movie real time: 400 sec.
(AVI)

Movie S2 **Poleward tubulin flux during metaphase of *D. melanogaster* S2 cells stably expressing EGFP-tubulin.** Two thin rectangular region of interest (ROI) were drawn on each half spindle and bleached by a 488 nm wavelength laser beam at time 0. The speed of movement of the bleached regions was used to estimate the rate of tubulin poleward flux. From left to right: Control mock dsRNAi treated cell; KLP10A KD cell; KLP10A-WT rescue cell; KLP10A-KT2M (KLP10A Kin-Tub-2 mutant) rescue cell. Total movie real time: 60 sec.
(AVI)
